# Evaluation of pharmacokinetic interactions of amoxicillin with ranitidine in healthy human volunteers of Karachi, Pakistan

**DOI:** 10.1371/journal.pone.0267791

**Published:** 2022-05-24

**Authors:** Shaheen Perveen, Shahnaz Gauhar, Rabia Ismail Yousuf, Huma Ali, Farya Zafar, Anab Fatima Sheikh

**Affiliations:** 1 Faculty of Pharmacy, Department of Pharmaceutics, University of Karachi, Karachi, Pakistan; 2 Department of Pharmaceutics, Institute of Pharmaceutical Sciences, Jinnah Sindh Medical University, Karachi, Pakistan; 3 RAK College of Pharmaceutical Sciences, RAK MHSU, Ras Al Khaimah, UAE; 4 College of Pharmacy, Dow University of Health Sciences, Karachi, Pakistan; Central University of Rajasthan, INDIA

## Abstract

Polypharmacy may be considered as the customary practice to provide optimum care services to patients but inter resulted in augmented probability of multiple drug interaction. Keeping in view the importance of drug interaction possibility, this study was designed to evaluate the effect of ranitidine on pharmacokinetics of amoxicillin in the local population of Karachi, Pakistan. Amoxicillin and ranitidine are the most commonly prescribed drugs to treat duodenal ulcer caused by *Helicobacter pylori*. The current investigation was carried out as a single center, open label, two phase, single dose, randomized way in cross over manner to evaluate the potential of pharmacokinetic interaction among amoxicillin formulation and ranitidine in adult healthy male volunteers. Post dosing blood samples were collected at multiple time points that are 0.5, 1, 1.5, 2, 3, 4, 6, 8 and 10 hours after administering amoxicillin 250mg capsule with and without ranitidine. For estimation of amoxicillin concentration in plasma, an HPLC method was developed and validated. The solvent system consisted of 0.025M phosphate buffer: acetonitrile (94:6 v/v). C18 column was employed with a flow rate of 1.0 ml/minute and at 230nm. A linear pattern with a correlation coefficient of 0.999 in the concentration ranges of 25μg/mL to 0.097μg/mL for amoxicillin and 25μg/mL to 0.048μg/mL for ranitidine was observed. Amoxicillin retention time was about 8 minutes and ranitidine retention time was around 12 minutes. Amoxicillin levels were computed and the concentrations were applied to calculate the pharmacokinetic parameters. Pharmacokinetic parameters were estimated by Kinetica TM 4.4.1 (Thermo Electron Corp. USA). The analysis of variance (two way) and t test (two one sided) were applied on log transformed pharmacokinetic parameters of amoxicillin. The T_*max*_ was determined between amoxicillin alone and amoxicillin with ranitidine by Friedman test. The 90% confidence interval values for C_*max(calc)*_ (0.687–0.743) and T_*max(calc)*_ (1.148–1.742) for amoxicillin with or without ranitidine were not found within the FDA acceptable limits of 0.8–1.25. Study demonstrated the significant reduction in peak plasma levels of amoxicillin in presence of ranitidine. It is advisable to administer both drugs with time interval to avoid such interactions and increases in the bactericidal efficacy of amoxicillin.

## 1. Introduction

In order to treat a patient with a single disease or multiple diseases, different drugs are usually administered to the same patient. During hospitalization, inpatients usually receive multiple drugs due to various comorbid situations including cardiovascular, gastrointestinal or neurological diseases, dermatological disorders, hepatic or renal impairment or irrational use of medicines. Drug interaction may occur during multiple drug therapies [1–3] and one of the most common cause of drug-drug interaction is medication error that results in adverse drug reactions.

In relation to drug-drug interactions, it is crucial to know whether co-administration of another drug therapy influences the effectiveness and safety of one drug therapy or not. A drug-drug interaction arises when one drug’s pharmacodynamics and/or pharmacokinetics are changed by another drug. Co-administration of two drugs may result in one of the most precarious forms of drug interactions. Reported results indicate that drug-drug interaction can affect various aspects of pharmacokinetics. Most common evidences were found to be alteration in the absorption (altered gastric pH, gastric emptying time etc.) [4–6], distribution (displacement of drug from its protein binding sites etc.) [7], metabolism (induction or inhibition of enzymes etc.) [8] and excretion (alter tubular reabsorption etc.) [9] of drugs were found. These drug interactions may reduce or increase the therapeutic efficacy and/or may be responsible for adverse drug reactions [10]. In order to maintain the importance of safe and effective therapeutic management, drug-drug interactions must be identified.

Amoxicillin (semi synthetic penicillin) is a frequently prescribed drug due to its good oral absorption. Amoxicillin is a broad spectrum β-lactam antibiotic that is commonly used in clinical practice [11–16]. Amoxicillin destroys bacteria by preventing them from making cell walls. The inability to build a cell wall will lead to cell breakdown, which destroys bacteria. It is used to treat infections, including chronic bronchitis flare-ups, bone and joint infections, biliary tract infections and actinomycosis. It has a high oral absorption rate of over 90% and a high overall plasma concentration [13,17]. The peak plasma concentration from 250 mg amoxicillin capsules was achieved in the range of and 3.5 to 5.0μg/mL. The reported half-life of amoxicillin in healthy volunteers is 1 hour. The plasma protein binding of amoxicillin is 17% and its distribution is rapid [12,15]. When amoxicillin is administered with diclofenac sodium, the bioavailability of amoxicillin is reduced [18].

Ranitidine is a H2 receptor blocker, it is the most frequently prescribed drug and is used to treat gastro esophageal reflux disease and gastric or duodenal ulcer [19]. It achieves peak plasma concentration in 2–3 hours with rapid absorption. The elimination half-life of ranitidine is about 2–3 hours. Ranitidine absorption is not significantly affected by food. Its bioavailability is near 50% and plasma protein binding is 15%. Main applications of ranitidine include treatment of peptic ulcer, gastro esophageal reflux disease, Zollinger-Ellison syndrome, stress ulceration and persistent dyspepsia. It shows pharmacokinetic interaction with metoclopramide.

Pletz *et al*. also reported pharmacokinetic interaction of ranitidine with ABT-773 on 12 healthy volunteers [20,21]. The adverse effects of ranitidine are minimal, such as headache, constipation, diarrhea, fatigue and drowsiness [22].

Different studies reported the occurrence of gastroduodenal ulcer in local population of Karachi, Pakistan in association with *Helicobacter pylori* infection [23,24]. A combination of ranitidine or proton pump inhibitor with antimicrobial agents preferably amoxicillin, clarithromycin or metronidazole can be used to heal ulcer and prevent relapse [25]. In Pakistan, amoxicillin is usually used in combination with ranitidine to treat duodenal ulcers. In one pharmacokinetic study, Yusuke Endo *et al*. evaluated the pharmacokinetic interaction of ranitidine 300mg tablets and daijokito (Kampo medicine) on seven healthy volunteers [26].

Hence, in this study the pharmacokinetic (PK) interaction between Amoxicillin 250 mg capsules and ranitidine 300mg tablets has been studied in a randomized, two phasic, crossover, single dose study following FDA guidelines in local population.

## 2. Materials and methods

### 2.1 Materials

Amoxicillin trihydrate capsules (250mg) and Ranitidine hydrochloride tablets (300mg) manufactured by GlaxoSmithKline Pakistan Ltd. were purchased from the community market. Crucial information like manufacturing and expiry dates were noted along with the batch number. Ranitidine hydrochloride and amoxicillin trihydrate reference standards used were gifts by Central Drug Laboratory, Karachi, Pakistan and Indus Pharma (Pvt) Limited respectively. Disodium hydrogen phosphate, O*rtho*phosphoric acid, acetonitrile (HPLC grade) and methanol were of HPLC grade, Merck Germany. Deionizer; Elga, High Wycombe, England. Borosilicate Glassware (Pyrex, England) was used. HPLC system was coupled with LC-10 AT VP pump, detector specifications were SPD-10 A UV detector and Class GC software from Shimadzu were used, system efficiency was also optimized with guard column (Merck, Germany). Method developmenta was carried out using Purospher star RP-18 (250mm, 5 μm, × 4.6 mm) column, a highly sensitive analytical balance (Mettler Toledo B204-S, Switzerland), centrifuge (Hereues, Osterode, Germany), pH meter (370 pH meter, Jenway, Europe), microliter syringe (Hamilton, Switzerland), filtration assembly (Sartorious, Germany), vortex mixer (Whirl Mixer, England), swinney membrane filter (Millipore, England), sonicator (LC20H) and Millipore filtration assembly (Millipore, England) were used.

In the present study Amoxicillin 250 mg capsules sold under the name of Amoxil and ranitidine 300mg tablets sold under the name of Zantac were selected as reference products for the PK interaction study as both are the innovator products of GlaxoSmithKline, Pakistan Ltd.

### 2.2 Methods

#### 2.2.1 Pharmacokinetic (PK) interaction study

A PK interaction study was conducted in a hospital setting to estimate various pharmacokinetic parameters of both products and then variables were compared to estimate the possibility of PK interaction between Amoxicillin 250 mg capsules and ranitidine 300 mg tablets, selected as reference products. Batch number, date of manufacturing and expiry are mentioned in Table 1. The aim of this research was to see whether there was a pharmacokinetic drug interaction between oral amoxicillin and ranitidine doses. Various guidelines based on European region provided for the sample size requirement during bioequivalence studies with minimum of 12 subjects [27]. Generally, the sample size of bioequivalence studies are mainly calculated on the basis of power calculation which mainly consider coefficient of variation of intrasubject variability and the matrices of T/R ratio. However, as T/R ratio is not clear before the study, it is likely considered that there will not be a difference of more than 55 between the treatments [28]. A crossover design using within-subject variance rather than between-subject variance was used with a sample size of 12 volunteers and was estimated to have at least 90% power (α = 0.05) to distinguish mean percentage variation of 20% for AUC of amoxicillin and ranitidine AUC respectively.

**Table 1:** Physicochemical attributes of amoxicillin and ranitidine.

Product Name	Batch No.	Mfg. Date	Expiry Date	Weight variation N = 20 Mean±SD	Hardness N = 20 Mean±SD	Disintegration N = 6 Mean±SD	Dissolution N = 6 Mean±SD	Content uniformity N = 20 Mean±SD	HPLC Assay N = 20 Mean±SD
**Amoxil**	2AFBA	Feb-13	Feb-15	352.73±1.89	N/A	N/A	96.88±2.19	99.69±0.076	98.98±0.699
**Zantac**	2ZCAC	Jun-12	Jun-15	490.20±1.63	16.015±0.4	6.011±0.17	100.88±1.73	98.22±1.732	99.33±0.519

N/A = Not Applicable.

In the present study, twelve healthy male volunteers having code as V1 –V12 with age group between 21–28 years (mean age was 23.5 years), weight between 55–74 kg (mean weight was 64.33 kg) and height between 5.4–5.9 ft.in (mean height was 5.50 ft.in) were selected for the present study (S1 Table in S1 File). All vital signs, medical history, physical examinations and essential laboratory tests were obtained from the volunteers. Subjects whose clinical results were not within the specified limits were excluded from the study. All the volunteers were excluded from the study if they met the following criteria: smoked 10 or more cigarettes per day, ingested nicotine products or consumed alcohol, were involved in any pharmacokinetic study within the past three months, had an infection two weeks before the study, had a severe disease, or donated blood within the last thirty days. The volunteers were not allowed to take any beverages including orange juice, grape fruit juice, coffee and chocolates, 48 hours before and during the study.

The present study protocol was accepted by the Ethical Review Committee (ERC) of Ziauddin University, Karachi, Pakistan (0440612SPPHAR). Following ICH guidelines, all the subjects were notified about the possible events of the present study. A written consent in English and in local language (Urdu) was signed by all participants involved in the study. Volunteers were allowed to leave the study at any time without difficulty, however all volunteers were able to complete the study duration.

#### 2.2.2 Study design

The present PK interaction study was designed as a randomized, two phasic, crossover, single dose. Before the drug administration, all volunteers were asked to fast for 10 hours. The Amoxicillin 250 mg capsules were coded as “A”, and co-administration of Amoxicillin 250 mg capsules and ranitidine 300mg tablets were coded as “B”. The present study was conducted in two phases. Each subject randomly received A and B with 240 ml of drinking water following fasting for an overnight period. One week wash out period was given in between the doses (S2 Table in S1 File). Volunteers were required not to eat anything for two hours after receiving the drugs. S2 Table in S1 File indicates the order in which items were handled. At 0 hour, a blood sample was taken before the dose was administered, and at 0.5, 1, 1.5, 2, 3, 4, 6, 8, and 10 hours after the dose was administered, blood samples were taken. The blood samples were moved into heparin containing centrifuge tubes. To isolate plasma, blood samples were centrifuged at 4000 rpm for 8 minutes. Before analysis, plasma samples were frozen at -20°C in sterile glass tubes.

The validated bio-analytical HPLC method was used to analyze plasma samples for the estimation of Amoxicillin in human plasma. For the estimation of amoxicillin in human plasma, an RP-HPLC method was developed. 0.025M phosphate buffer: acetonitrile (94:6 v/v) with pH 3.1 was used as the mobile process. For separation, C18 column was used with a flow rate of 1.0 ml/minute and at a wavelength of 230nm. ICH Q2-B guidelines were taken into consideration for validation of the bioanalytical method.

All the volunteers were assessed in order to determine any adverse effects related to the drug by physical examination and other clinical tests. Volunteers were encouraged to immediately notify in case of any unusual event. Complete physical examinations and vital signs determinations were carried out at screening period and on study days of each treatment phase. Subjects were asked for any significant signs of GI disturbances, pain or any noticeable conditions.

In the present study non-compartmental analysis using Kinetica® version 4.4.1, Thermo electron Corp., USA has been applied for the estimation of various PK parameters and variables including T_*max*_, C_*max*_, AUC_*0-α*_, AUC_*0-t*_, λ_z_, T_*1/2λz*_, AUMC and MRT. The pharmacokinetic data was fitted in one compartment oral model. Different parameters like sequence effect, subject effect, period effect were calculated by Latin ANOVA (two–way) using Kinetica® version 4.4.1. ANOVA was applied. The PK variables were analyzed on log transformed pharmacokinetic data of Amoxicillin (alone) and Amoxicillin and ranitidine (in combination). The 90 percent CIs were determined using the acceptance limits of 0.8–1.25 and 0.8–1.2 respectively, based on differences in geometric mean values of log transformed results.

## 3. Results and discussion

The pharmacokinetic parameters of amoxicillin were calculated and compared to those obtained in combination with ranitidine to see if there was any pharmacokinetic interaction. An analytical method validation was performed for the determination of plasma drug concentration profile under the guidelines of ICHQ2B. Intraday and interday accuracy and precision were found within acceptable limit and method showed the linearity with a correlation coefficient of 0.999 (S1 and S2 Figs in S1 File) in the concentration ranges of 0.097μg/mL to 25μg/mL for amoxicillin and 0.048μg/mL to 25μg/mL for ranitidine (S3 and S4 Tables in S1 File). Amoxicillin and ranitidine retention times were about 8 and 12 minutes respectively. S5 Table in S1 File shows bioanalytical method validation of ranitidine and amoxicillin simultaneously. Plasma drug stability was also found within acceptable limits. (S6 Table in S1 File). Amoxicillin 250 mg capsules (Amoxil) and ranitidine 300 mg tablets (Zantac) were selected as reference products. Table 1 indicates some physicochemical attributes of the studied drugs.

**Fig 1:**
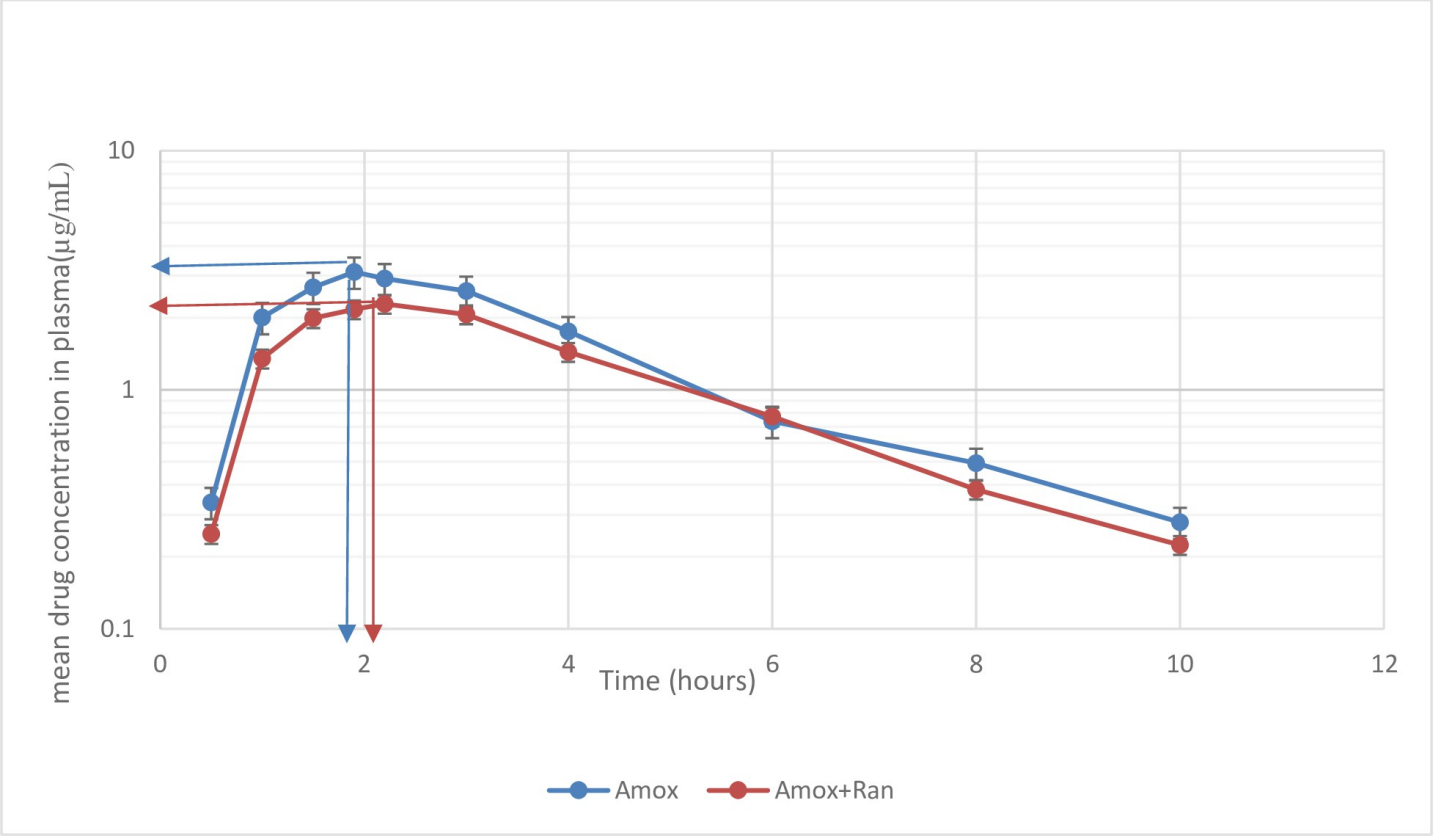
Mean plasma concentration of amoxicillin alone and amoxicillin with ranitidine.

For pharmacokinetic analysis of amoxicillin, PK/PD software, Kinetica® version 4.4.1, Thermo electron Corp., USA was used. These variables were statistically tested to see whether there was any pharmacokinetic interaction between the two products. To compare the pharmacokinetic parameters of amoxicillin given alone and in combination with ranitidine, Analysis of variance (ANOVA) latin square was used. To compare T_*max*_ values, non-parametric Friedman was used using SPSS Version 20 programme. By considering the acceptance limits of 0.8–1.25 and 0.8–1.2, respectively, at 90 percent confidence interval (CI), p value of less than 0.05 was measured statistically significant to note the geometric mean value differences of log transformed results. Fig 1 indicates the mean plasma concentrations of amoxicillin alone and in combination with ranitidine.

The geometric mean values of measured C_*max*_ were 3.113± 0.064 μg/mL for amoxicillin given alone and 2.288± 0.043 μg/mL when it is given with ranitidine. When amoxicillin was given with or without ranitidine, there was a very large difference in measured C_*max*_ (p = 0.0008). Non compartmental analysis exhibited that the geometric mean of observed C_*max*_ for amoxicillin alone was 3.099±0.141μg/mL and 2.477± 0.190 μg/mL for amoxicillin administered with ranitidine. When p value is equal to 0.015, there was a substantial difference between amoxicillin administered alone and with ranitidine. For amoxicillin alone, the geometric mean for calculated T_*max*_ was 1.912± 0.019 hrs and 2.228± 0.031 hrs for amoxicillin given with ranitidine. The 90 percent confidence interval was 114.8 to 117.4 percent. There was a significant difference in T_*max*_ of amoxicillin in the presence and absence of ranitidine (p = 0.0001). AlGaai *et al*. conducted a bioequivalence study on two amoxicillin formulations and found T_*max*_ values of 1.92 ± 0.76 and 2.02 ± 0.62 hr for test and reference formulation respectively [29]. For amoxicillin alone, the geometric mean of AUC_*0-t*_ was 12.582±0.272 mg/L.hr and for amoxicillin given with ranitidine, it was 10.611±0.359 mg/L.hr. A highly significant difference in AUC_0-t_ of amoxicillin administered alone and in presence of ranitidine with p value of 0.0033. The geometric mean of AUC_*total*_ for amoxicillin given alone and coadministered with ranitidine were 12.783±0.301 mg/L.hr and 11.192±0.182 mg/L.hr correspondingly. For amoxicillin, the geometric mean of clearance was 19.817±0.394 L/hr and 23.524±0.411 L/hr for amoxicillin plus ranitidine. In presence of ranitidine, amoxicillin clearance was significantly reduced (p = 0.001). Amoxicillin’s geometric mean volume of distribution (V_*c*_) was 31.383±0.604 L, and amoxicillin’s volume of distribution in the presence of ranitidine was 46.137±0.503 L. The volume of distribution (V_*c*_) of amoxicillin in presence or absence of ranitidine was significantly different (p = 0.0007). ANOVA was used to test the log transformed data of amoxicillin, with less than 0.05 p value deemed statistically relevant at 90% confidence interval and at 0.8–1.2 acceptance limit. No significant effect of subject, time or sequence on measured C_*max*_, observed C_*max*_ or T_*max*_ of amoxicillin was found as p values were more than 0.05. When amoxicillin was co-administered with ranitidine, the AUC_*0-t*,_ AUC_*last*_ and AUC_*total*_ values were significantly modified (p < 0.05), but there was no significant impact of covariables. The 90 percent confidence intervals on log transformed C_*max*_ values of amoxicillin alone and in presence of ranitidine were found to be beyond the acceptance limits of 0.8–1.2 and 0.8–1.25 respectively (Tables 2 and 3).

**Table 2:** Mean compartmental and non-compartmental pharmacokinetic parameters of amoxicillin 250 mg capsule after administration to healthy volunteers alone and in combination with ranitidine 300mg tablet.

Values of amoxicillin capsule 250mg									
**Compartmental** **Parameters**	**C**_***max calc***_	**T**_***max calc***_	**K**_***el***_	**AUC**	**K**_***a***_	**Cl**	**V**_***c***_	**T**_***1/2 Kel***_	**T**_***1/2 Ka***_
	**μg/mL**	**hr**	**hr** ^**-1**^	**mg/L.hr**	**hr** ^**-1**^	**L/hr**	**L**	**hr**	**hr**
**Mean**	3.113	1.912	0.636	12.582	0.715	19.817	31.383	1.088	0.973
**SD**	0.064	0.019	0.008	0.272	0.015	0.394	0.604	0.019	0.016
**% CV**	2.065	1.014	1.334	2.164	2.072	1.99	1.924	1.745	1.649
**Values of amoxicillin capsule 250mg along with ranitidine tablet 300mg**									
**Mean**	2.288	2.228	0.511	10.611	0.653	23.524	46.137	1.357	1.069
**SD**	0.043	0.031	0.011	0.359	0.012	0.411	0.503	0.019	0.016
**%CV**	1.897	1.382	2.092	3.386	1.801	1.747	1.090	1.403	1.476
**Values of amoxicillin capsule 250mg**									
**Non compartmental Parameters**	**AUMC**	**MRT**	**λ**_***z***_	**AUC**_***last***_	**AUC**_***tot***_	**AUMC**_***tot***_	**V**_***z***_	**T**_***1/2 λz***_	
	**mg/L.(hr)** ^**2**^	**hr**	**L/hr**	mg/L*h	mg/L*h	mg/L*(h)^2^	**L**	**Hr**	
**Mean**	37.52	3.391	0.464	12.552	12.783	43.344	42.139	1.101	
**SD**	0.974	0.014	0.006	0.297	0.301	1.019	1.128	0.041	
**%CV**	2.595	0.427	1.214	2.367	2.354	2.352	2.678	3.699	
**Amoxicillin capsule 250mg with ranitidine tablet 300mg**									
**Mean**	37.131	4.284	0.317	10.445	11.192	47.105	71.094	1.358	
**SD**	1.357	0.088	0.011	0.416	0.182	0.932	2.911	0.059	
**% CV**	3.655	2.051	3.57	3.981	1.624	1.979	4.095	4.352	

**Table 3:** Statistical assessment of amoxicillin pharmacokinetic interaction with ranitidine.

Pharmacokinetic Parameters			Log- Transformed Data		
	Amoxicillin alone (Mean±SD)		*Geomean Ratio (Test/Reference)*	*90%*	P value
		Amoxicillin + Ranitidine (Mean±SD)		*Confidence Interval*	*** significant difference
**C**_**max calc (**_**μg/mL)** ↓	3.113±0.064	2.288±0.043	0.7151	0.68772, 0.74369	2.503e-008 ***
**T**_***max calc***_ (hr)↑	1.912±0.019	2.228±0.031	1.1611	1.1483, 1.1742	3.222e-010 ***
**AUC**_***0-∞***_ **(mg/L.hr**)*↓*	12.582±0.272	10.611±0.359	0.8404	0.82061, 0.86087	1.23e-007 ***
decrease ↓					
increase ↑					

A substantial difference in amoxicillin T_*max*_ values in the presence and absence of ranitidine was shown by Friedman test (p = 0.001) (Table 4).

**Table 4:** NPar and FRIEDMAN Analysis for differentiation of T_max_ for amoxicillin alone and in combination with ranitidine.

NPar Tests				
**Parameters of Descriptive Statistics**	N	Values of Percentiles		
		25^th^ percentile	50th (Median) percentile	75^th^ percentile
**T**_**max**_**(**Amoxicillin+Ranitidine)	12	2.18150	2.22600	2.25225
**T**_**max**_(Amoxicillin)	12	1.89625	1.91000	1.93050
**Test of Friedman**				
**Ranks**			**Values (Mean)**	
**T**_**max**_ (Amoxicillin+Ranitidine)			2.00	
**T**_**max**_ (Amoxicillin)			1.00	
**Test Statistics** ^**a**^				
Number N			12	
Values of Chi-Square			12.00** (insignificant)	
df			1	
Asymp. Sig.			0.001	

In current examination, amoxicillin mathematical mean elimination rate constant (K_*el*_) and end half-life (T_*1/2Kel*_) managed alone were discovered to be 0.636 ± 0.008 hr^-1^ and 1.088 ± 0.019 hr while when given with ranitidine; K_*el*_ was decreased to 0.511 ± 0.011 hr^-1^ and T _*½ Kel*_ was expanded to 1.357 ± 0.019 hr. The elimination half-life and rate constant of amoxicillin was markedly altered when administered with ranitidine (p = 0.003). Guoxin *et al*., 2002 announced that the elimination half-lives calculated by amoxicillin administration of test and reference formulation were 1.09±0.22 hr and 1.43±0.44 hr respectively [30]. K*el* and half life are dependent upon clearance and volume of distribution. However, it would be unreasonable to make any assumptions about the parameters of a drug solely on the basis of its half life. For amoxicillin, the geometric mean of absorption rate constant was observed to be 0.715 ± 0.015 hr^-1^ when given alone and 0.653 ± 0.012 hr^-1^ when given along with ranitidine. The values for amoxicillin absorption half-life were 0.973 ± 0.016 hr and 1.069 ± 0.016 hr when amoxicillin given alone and in combination with ranitidine respectively. There was a significant effect exhibited on amoxicillin absorption rate constant and amoxicillin absorption half-life when given with ranitidine with p value of 0.0004. (Tables 4 and 6).

The pharmacokinetics evaluation was carried out statistically between amoxicillin alone and in presence of ranitidine through Latin Square (Kinetica^TM^ User Manual, 2005). Drug interaction was considered significant if the difference in amoxicillin parameters did not fall between 0.80 and 1.25 using 90% CI after log transformation of data. The geometric mean ratios of C_*max*_, T_*max*_, AUC_*tot*_. AUC_*0-t*_. AUC_*0-∞*_, T_*1/2Kel*_ and MRT of log-transformed data were calculated as 0.715, 1.161, 0.870, 0.831, 0.840, 1.233 and 1.263 respectively (Table 5). The effect of period, sequence, subject and treatment were also evaluated.

**Table 5:** Log transformed pharmacokinetics parameters of amoxicillin alone and in combination for inter and intra subject variability.

Factors	C_*max*_ (μg/mL)	AUC_*tot*_ (mg/L*h)	AUC_*0-α*_ (mg/L*h)	AUC _*0-t*_ (mg/L*h)	T _*1/2 Kel*_ (hr)	MRT (hr)	T_*max*_ (hr)
GMR*	0.715	0.870	0.840	0.831	1.233	1.263	1.161
Test power	Near to 0	1	Near to 1	Near to 1	Near to 1	Near to 1	Near to 1
%RSD**	0.054	0.016	0.013	0.015	0.191	0.012	0.020
RMSE***	0.052	0.039	0.032	0.038	0.038	0.016	0.015
***P values***							
Period	0.2776 NS	0.2638 NS	0.1361 NS	0.1965 NS	0.3068 NS	0.4218 NS	0.5984 NS
Subject	0.8455 NS	0.8372 NS	0.7705 NS	0.8739 NS	0.4223 NS	0.8266 NS	0.5928 NS
Treatment	2.503e-008 ***	6.307e-006 ***	1.23e-007 ***	3.69e-007 ***	9.953e-008 ***	1.164e-011 ***	3.222e-010 ***
Sequence	0.7042 NS	0.9607 NS	0.8001 NS	0.8414 NS	0.2835 NS	0.3554 NS	0.5607 NS
***F- values***							
Period	1.31822	1.40154	2.6269	1.91517	1.16002	0.701601	0.295836
Subject	0.514052	0.525854	0.617318	0.471961	1.13549	0.540747	0.858795
Treatment	241.179	73.719	172.929	137.012	180.798	1152.32	588.687
Sequence	0.152614	0.00255379	0.0676561	0.0421862	1.28434	0.939104	0.362193

GMR*: Geometric mean Ratio; RSD**: Relative standard deviation; RMSE***: Root mean square error.

Table 6 includes a list of the adverse events. No variations in the rates of adverse drug outcomes between treatment groups were observed, and no one dropped out because of the side effects. Moreover, no serious adverse effects were found throughout the course of study, and if any observed, all were moderate. It was also observed that common side effects associated with amoxicillin like nausea, abdominal pain and related gastric issues were reduced in patients treated using combination therapy with ranitidine. Furthermore, no clinically meaningful deviations in any of the laboratory parameters were found. No clinically important changes in vital sign values were observed on any laboratory parameter or at any stage in a physical examination.

**Table 6:** Adverse events reported by subjects.

Reported Events	Amoxicillin	Amoxicillin with ranitidine
Nausea	2	1
Headache	0	1
Diarrhea	1	0
Abdominal Pain	1	0
Vomiting	1	0
Flatulence	2	1
Total	7	3

*given values are real number of adverse events on the basis of individual subject reporting. Statistically insignificant differences were found between treatments.

## 4. Conclusion

The present study reveals the significant pharmacokinetic interaction between amoxicillin and ranitidine. A noteworthy change in important pharmacokinetic parameters was found with decline in levels of AUC_*tot*_, T_*max*_, and AUC_*last*_. Whereas, an augmented effect in T_*1/2Kel*_, T_*1/2 Ka*_, V_*c*_, and MRT was observed. In our study, these parameters have significantly changed. AUC and T_*max*_ values are found within the 80–125% range but with increase in T_*max*_ and decrease in AUC, however, C_*max*_ values were declined significantly with GMR of 0.71. It has been suggested that amoxicillin and ranitidine should not be co-administered simultaneously to have a proficient bactericidal outcome. Moreover, this study defines the significant drug interaction in various pharmacokinetic parameters. However, this study is limited with respect to multicenter aspect and single dosing based pharmacokinetic evaluations. As different doses present an additional variable which increases the number of participants and samples required to develop the model. In future, Fed state pharmacokinetic interaction may also be determined. Besides, this study may be performed in different age groups, population with comorbid conditions to develop more generalized effects of interaction. Moreover, the clinical significant evaluation of the observed pharmacokinetic interaction needs further research.

## Supporting information

S1 File Raw data. (XLSX)
